# Implicit causality biases and thematic roles in American Sign Language

**DOI:** 10.3758/s13428-021-01561-1

**Published:** 2021-03-29

**Authors:** Anne Therese Frederiksen, Rachel I. Mayberry

**Affiliations:** 1 Department of Linguistics, University of California, San Diego, 9500 Gilman Drive, MC 0108, La Jolla, CA 92093-0108 USA; 2 Present Address: Department of Language Science, University of California, Irvine, Social Science Plaza B #2239, Irvine, CA 92697-5100 USA

**Keywords:** Implicit causality bias, American Sign Language, Psychological verbs, Thematic roles, Body-anchoring

## Abstract

Implicit causality (IC) biases, the tendency of certain verbs to elicit re-mention of either the first-mentioned noun phrase (NP1) or the second-mentioned noun phrase (NP2) from the previous clause, are important in psycholinguistic research. Understanding IC verbs and the source of their biases in signed as well as spoken languages helps elucidate whether these phenomena are language general or specific to the spoken modality. As the first of its kind, this study investigates IC biases in American Sign Language (ASL) and provides IC bias norms for over 200 verbs, facilitating future psycholinguistic studies of ASL and comparisons of spoken versus signed languages. We investigated whether native ASL signers continued sentences with IC verbs (e.g., ASL equivalents of ‘Lisa annoys Maya because…’) by mentioning NP1 (i.e., Lisa) or NP2 (i.e., Maya). We found a tendency towards more NP2-biased verbs. Previous work has found that a verb’s thematic roles predict bias direction: stimulus-experiencer verbs (e.g., ‘annoy’), where the first argument is the stimulus (causing annoyance) and the second argument is the experiencer (experiencing annoyance), elicit more NP1 continuations. Verbs with experiencer-stimulus thematic roles (e.g., ‘love’) elicit more NP2 continuations. We probed whether the trend towards more NP2-biased verbs was related to an existing claim that stimulus-experiencer verbs do not exist in sign languages. We found that stimulus-experiencer structure, while permitted, is infrequent, impacting the IC bias distribution in ASL. Nevertheless, thematic roles predict IC bias in ASL, suggesting that the thematic role-IC bias relationship is stable across languages as well as modalities.

## Introduction

The present study investigates how implicit causality (IC) biases are distributed in American Sign Language (ASL) verbs and provides norming data that can be applied in future studies. IC biases are at the basis of many psycholinguistic processes, but at this time, no published norms exist for IC biases in ASL or any other sign language. The present study also examines potential modality differences in how thematic roles relate to IC bias in ASL compared to English.

The prominent role of implicit causality (IC) biases in psycholinguistic research is due to their role in many processes in language comprehension, such as reading time, sentence processing and most notably in the resolution of pronominal anaphora (Garvey et al., 1976). Due to IC biases, sentences such as (1) and (2) do not cause comprehension problems, despite the fact that the pronoun is temporarily ambiguous. In (1) the verb ‘impress’ creates a bias towards the subject ‘Lisa’, whereas ‘hate’ in (2) creates a bias towards the object ‘Maya’. The pronoun is then interpreted in line with the bias, effectively resolving its ambiguity.

1. Lisa_i_ impresses Maya_j_, because she_i_ …
2. Lisa_i_ hates Maya_j_, because she_j_ …

Because of their effect on language processing, IC biases play a role in theories of discourse coherence (Pickering & Majid, 2007; Kehler, Kertz, Rohde & Elman, 2008; Kehler & Rohde, 2019). Researchers have asked whether these biases are the same across languages and cultures, and whether they result from linguistic processes or cognitive universals (Ferstl et al., 2011; Goikoetxea et al., 2008; Hartshorne, 2013; Hartshorne et al., 2013; Rudolph & Forsterling, 1997).

IC biases are closely related to semantic properties of the verb along with the surrounding discourse structure (discussed in more detail below in section 2.1; Brown & Fish, 1983a, 1983b; Au, 1986; Rudolph & Försterling, 1997; Ferstl et al., 2011, Hartshorne & Snedeker, 2012). Specifically, there is a link between thematic roles and the direction of bias in IC verbs (Au, 1986; Brown & Fish, 1983a, 1983b; Crinean & Garnham, 2006; Rudolph & Forsterling, 1997), such that thematic roles roughly predict IC biases. The notion of thematic role captures the semantic relationship between a verb and its noun phrase argument. For example, the verb ‘walk’ takes an agent thematic role (i.e., the person walking), the verb ‘dream’ takes an experiencer thematic role (the person dreaming), and the verb ‘love’ takes both an experiencer and a stimulus thematic role (i.e., the person feeling the love and the person being loved). In transitive verbs, the ordering of thematic roles reflects their mapping onto syntactic roles: in stimulus-experiencer verbs (e.g., ‘annoy’), the stimulus corresponds to the noun phrase argument appearing first in the sentence (NP1), whereas in experiencer-stimulus verbs (e.g., ‘love’), the stimulus corresponds to the noun phrase argument that appears second in the sentence (NP2). The type of thematic roles a verb has, and how these roles map onto the syntax affect the direction of the verb bias and therefore which argument will be re-mentioned: agent-patient (e.g., ‘kiss’) and stimulus-experiencer (e.g., ‘annoy’) verbs generally elicit re-mentions of NP1; agent-evocator (e.g., ‘blame’) and experiencer-stimulus (e.g., ‘love’) verbs generally elicit re-mentions of NP2.

IC biases are expected to behave similarly across languages because they are tied to verb semantics in this way. Based on the languages examined to date, it indeed appears that verb semantics predict IC bias cross-linguistically (Hartshorne et al., 2013; Bott & Solstad, 2014). However, it remains an empirical question as to whether implicit causality biases behave similarly across sensory-motor modalities, and whether their relation to thematic roles is the same in signed as in spoken languages. The answers to these questions have implications for the cross-linguistic validity of theories of discourse coherence and the role of IC biases in them. To date, IC biases have not been documented for any sign language. ASL has been shown to have semantic structures that are similar to those found in spoken languages (Kegl, 1990). Nevertheless, there are certain features of the visual-manual modality which could affect either the relationship between thematic roles and IC biases in signed as compared to spoken languages or lead to distributions of IC verbs with NP1- vs. NP2-biases that are different in the signed as compared to the spoken modality.

**Fig. 1:**
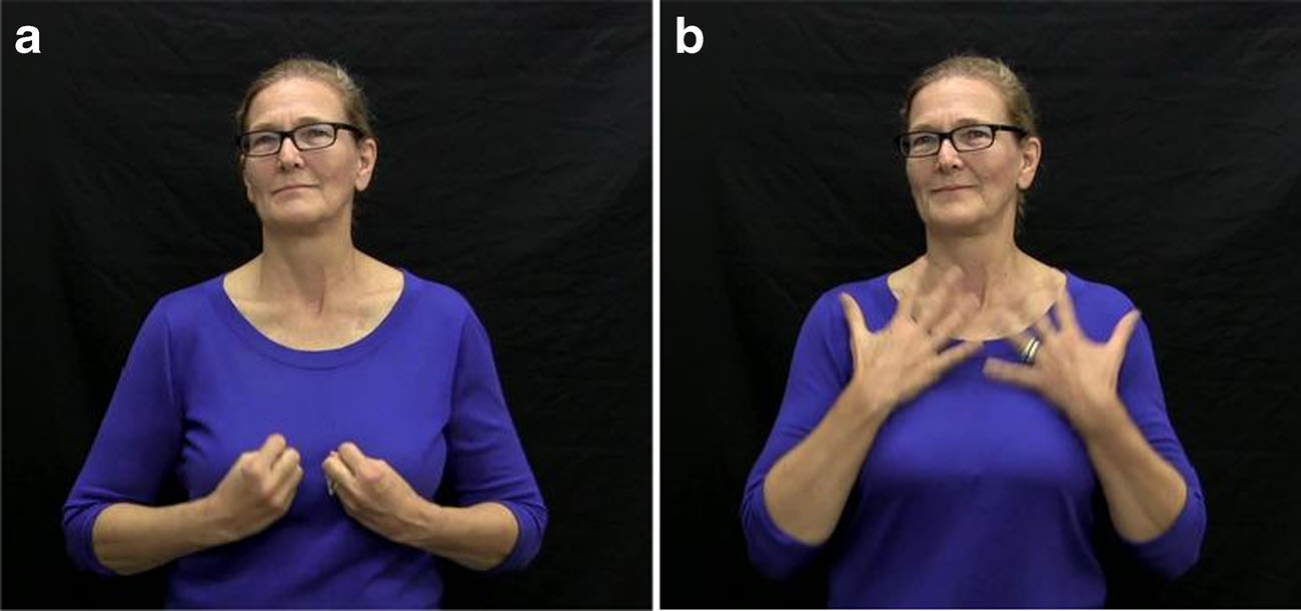
The body-anchored verb INSPIRE at the beginning (panel a) and end (panel b) of the sign

One such feature is *body-anchoring* (discussed in more detail in section 2.2.2), a phenomenon whereby some signs are obligatorily articulated on or near a particular part of the signer’s body, rather than in neutral signing space. For example, the sign ‘INSPIRE’ in Fig. 1 is a body-anchored sign, articulated by opening the hands and moving them upwards on the signer’s chest. In the present paper, we follow the convention from the sign linguistics literature of using capitalized words to represent sign glosses. Although English words are used for glosses, a sign’s meaning is not necessarily a translation equivalent of the English word in all linguistic contexts.

In verbs such as ‘INSPIRE’, the sign is often articulated at the place on the body where a represented action or emotion is (metaphorically) situated. In the case of ‘INSPIRE’, the sign represents a feeling (of inspiration) burning or spreading like fire up through one’s chest. Because of this, the signer’s body tends to be interpreted as the experiencer in body-anchored verbs (Edge & Herrmann, 1977; Kegl, 1990), that is, in a verb such as ‘INSPIRE’, the signer’s body is the experiencer of the inspiration feeling.^1^ This could affect implicit causality biases in ASL in at least two ways. First, the phonological (articulatory) structure of the verb could heighten the prominence of the experiencer and thereby increase the likelihood for the experiencer argument rather than the stimulus argument to be re-mentioned in upcoming discourse.^2^ In this scenario, the experiencer would be favored for re-mention in body-anchored verbs irrespective of their semantic structure. This would mean that experiencer-stimulus verbs which are generally NP2-biased in spoken languages would be NP1-biased in sign languages. Conversely, stimulus-experiencer verbs, which are generally NP1-biased in spoken languages, would instead be NP2-biased sign language verbs where the articulation location involves body contact. Thus, body-anchoring might alter the relationship between thematic role structure and implicit causality bias direction in ASL verbs.

Another possibility is that body-anchoring does not affect the relationship between thematic role and IC bias, but instead only leads to fewer NP1-biased verbs, compared to spoken languages like English. Rather than elevate the prominence of the experiencer, body-anchoring might affect the permissible thematic role structure in sign languages, essentially permitting only experiencer-stimulus verbs and not stimulus-experiencer verbs (the details of this argument are discussed in more detail below in section 2.2). In support of this possibility, previous research has claimed that verbs with stimulus-experiencer structure (that is, verbs like ‘annoy’ in English) are largely or totally absent in a number of sign languages (for ASL, Edge & Herrmann, 1977; Kegl, 1990; Winston, 2013; Healy, 2015; for Sign Language of the Netherlands, Oomen, 2017; for Israeli Sign Language, Meir et al., 2007; for Greek Sign Language, Sapountzaki, 2005). In many spoken languages, verbs with stimulus-experiencer structure are not only biased towards NP1, but strongly so (Crinean & Garnham, 2006; Rudolph & Forsterling, 1997). Consequently, a possible absence of stimulus-experiencer verbs should affect the distribution of IC biases in ASL, leading to fewer NP1-biased verbs than in a language like English. Moreover, the average bias of ASL verbs with NP1-biases should be weaker than in spoken languages that have stimulus-experiencer verbs.

To date, however, no published norms exist for IC biases in ASL verbs. Considering the importance of IC biases for language processing, investigating their distribution and relationship with semantic structure, specifically thematic roles, is important for understanding the processes that affect discourse coherence in a sign language and for developing cross-linguistically and cross-modally valid theories. The growing interest in conducting psycholinguistic studies in signed languages further underscores the importance of understanding IC biases in signed verbs. Obtaining normative data for sign language IC verbs in comparison with the IC biases in the spoken languages surrounding them provides an invaluable tool for future research. The present study fills this gap in our knowledge by providing implicit causality norming for more than 200 ASL verbs. We also investigate whether the phenomenon of body-anchoring affects the relationship between IC biases and thematic roles, how thematic role is lexicalized in signed as compared to spoken languages, and how this might influence the distribution of verb biases in ASL.

## Implicit causality biases

Implicit causality (IC) refers to the phenomenon whereby language users implicitly ascribe the cause of an event or state to one of the nominal arguments of the verb (Garvey & Caramazza, 1974, but see also Hartshorne, 2013). For example, in (1) above it is assumed that something about Lisa is impressive to Maya. Conversely, in (2) the assumption is that Maya has done something detestable to cause Lisa to hate her. In both cases, one expects the discourse to continue with an explanation of why this is. Therefore, the discourse is likely to continue referencing the causally implicated referent (e.g., ‘Lisa impresses Maya, because she (=Lisa) has a stellar resume’). Because of the alternation between ascribing causation to the subject (as in (1)) or the object (as in (2)), IC verbs are classified as either subject-biased or object-biased, often called NP1- and NP2-biased verbs (Garvey & Caramazza, 1974). It is important to note, however, that IC biases in verbs exist on a continuum. This continuum ranges from fully biased towards the subject (NP1-biased) to fully biased towards the object (NP2-biased), with some verbs exhibiting stronger biases than others.^3^

IC biases play a role in many processes in language comprehension. Several studies have noted the so-called *congruency effect* (Stewart et al., 2000), namely that going against the direction of the verb bias leads to slower comprehension compared to following the verb bias. A bias-incongruent sentence is by no means ungrammatical, however. For example, the sentence ‘Kate praised Liam because she felt obliged to do so’ is grammatical despite the re-mention of NP1 in the context of an NP2-biased verb (Ferstl et al., 2011: 125). Yet, studies have shown that the bias-incongruence causes processing delays. This has been found in the context of pronoun interpretation (Caramazza et al., 1977; Ehrlich, 1980; Koornneef & van Berkum, 2006; McKoon et al., 1993) and timed reading tasks (Garnham & Oakhill, 1985).

A number of studies have examined IC biases in English (Ferstl et al., 2011; Garvey et al., 1976; Garvey & Caramazza, 1974, among others). It is known that IC verbs exist in many languages, across cultures and ages. Studies have examined IC biases in languages such as Cantonese (Brown & Fish, 1983a), German (Fiedler & Semin, 1988; Rudolph, 1997; Bott & Solstad, 2014), Dutch (Koornneef & van Berkum, 2006; Semin & Marsman, 1994), Italian (Franco & Arcuri, 1990; Mannetti & De Grada, 1991), Spanish (Goikoetxea et al., 2008), Finnish (Pyykkönen & Järvikivi, 2010), Mandarin, Russian, Japanese (Hartshorne et al., 2013), and Norwegian (Bott & Solstad, 2014). Overall, these studies have found that IC verbs in these languages pattern similarly to what has been found for English, though the strength of the biases varies (Hartshorne et al., 2013; Rudolph & Forsterling, 1997). As noted by Goikoetxea et al. (2008), this variation suggests that it is necessary to gather normative data specifically for any language in which IC biases are used as a controlled experimental variable. However, cross-linguistic comparisons of implicit causality (IC) verbs require not only norming data, but also an understanding of the source of IC biases. In the next section, we discuss in more detail the relationship between verb semantics and IC bias and the cross-linguistic work that has explored these phenomena.

### Implicit causality and verb semantics

One of the first studies to investigate the source of IC biases, Brown and Fish (1983b) found evidence that re-mention preferences could be partially predicted from the semantic class of a verb. They divided verbs into mental (or state) verbs vs. behavioral (or action) verbs, linking the semantic classes to thematic roles and making different predictions about bias for each type. The revised action-state taxonomy based on this work created a more fine-grained taxonomy (Rudolph, 1997; Rudolph & Forsterling, 1997). This taxonomy divides verbs into four categories, based on their thematic roles: agent-evocator, Example (3); agent-patient, Example (4); stimulus-experiencer, Example (5); and experiencer-stimulus, Example (6).

(3) Lisa blamed Matt, because he had sabotaged the group project.
(4) Lisa kissed Matt, because she was in love with him.
(5) Lisa annoyed Matt, because she never cleaned the kitchen.
(6) Lisa loved Matt, because he was a good person.

This work and subsequent studies have found that thematic roles roughly predict IC biases (Au, 1986; Brown & Fish, 1983a, 1983b; Crinean & Garnham, 2006; Rudolph & Forsterling, 1997). Large-scale normative studies have provided additional support for this relationship: Goikoetxea et al. (2008) tested 100 verbs in Spanish, and Ferstl et al. (2011) tested 305 verbs in English. Their studies showed that agent-patient and stimulus-experiencer verbs are generally NP1-biased, and agent-evocator and experiencer-stimulus verbs are generally NP2-biased. Some researchers, however, have raised questions about whether thematic roles are the best predictor of IC biases. For example, Pickering and Majid (2007) argue against the existence of the agent-evocator thematic structure. Bott and Solstad (2014) highlight how thematic roles do not explain why biases can vary within groups of verbs with the same thematic roles. Hartshorne and Snedeker (2012) argue that IC biases result from verb classes (such as proposed by Levin, 1993, and Levin and Rappaport Hovav, 2005). They consequently suggest that a more fine-grained semantic verb classification better predicts IC bias in general (Hartshorne & Snedeker, 2012; see also Hartshorne et al., 2015). Nevertheless, the thematic role approach and the verb class approach make the same predictions for psychological verbs: verbs with stimulus-experiencer structure (sometimes also referred to as experiencer-object verbs) are biased towards the subject, NP1, while experiencer-stimulus verbs (sometimes referred to as experiencer-subject verbs) are biased towards the object, NP2.^4^ In the present paper, the discussion of the relationship between IC bias and thematic roles focuses on psychological verbs, so we do not distinguish between the thematic role approach and the verb class approach.

The proposed relationship between verb semantics and IC biases is supported by the fact that studies of languages other than English and Spanish have verified the phenomenon (Norwegian and German: Bott & Solstad, 2014; Mandarin, Russian and Japanese: Hartshorne et al., 2013). Due to the cross-linguistic nature of this phenomenon, we expect the mapping of thematic roles onto syntactic ones to be an important predictor for how IC biases are distributed within a language. Differences in the relationship between thematic and syntactic roles could result in different bias distributions cross-linguistically. For example, the lexical meaning ‘to miss’ is lexicalized in French with an experiencer-object verb (‘manquer’) but with an experiencer-subject in English (Hartshorne et al., 2010). Similarly, some languages, such as Mandarin, Japanese, and Atsugewi, have many verbs lexicalized with experiencer-subjects (experiencer-stimulus verbs), but few or none with experiencer-objects (stimulus-experiencer verbs). In Mandarin, most stimulus-experiencer structures are expressed using periphrastic constructions, rather than morphologically simple lexical items. This means that sentences such as ‘Lisa makes Maya angry’ are preferred over sentences such as ‘Lisa angers Maya’ (Chen, 1996; see also Zhang, 2002). In Japanese (Hartshorne et al., 2010) and Atsugewi (Talmy, 2000), the base form of psychological verbs typically takes an experiencer-subject. To modify the verb to take an experiencer-object instead, speakers then add an affix typically analyzed as a causative. As a result, languages such as Japanese have very few morphologically simple verbs with experiencer-objects (5, compared to 74 experiencer-subject verbs, according to a survey by Hartshorne et al., 2010). This is in comparison to other languages with an abundance of (morphologically simple) psychological verbs with experiencer-objects, for example English (220, compared to 44 experiencer-subject verbs; see Levin, 1993).

To date, no study has examined the relationship between verb semantics and IC biases in signed languages. However, as the previous discussion explains, a low proportion of verbs lexicalized with stimulus-experiencer structure in ASL might lead to a low proportion of NP1-biased verbs. Alternatively, the phenomenon of body-anchoring in ASL might diminish the predictive power of thematic roles for IC bias direction. In the next section, we focus on previous findings related to realization of thematic roles in sign languages. These findings provide some expectations about IC biases in ASL and whether thematic roles should impact the distribution of biases in implicit causality verbs in the manual-visual modality.

### Transitivity and mapping between semantic and syntactic roles in sign languages

Sign language studies have claimed that the stimulus-experiencer structure is largely absent in psychological verbs in sign languages such as ASL, Sign Language of the Netherlands (NGT), Israeli Sign Language (ISL) and Greek Sign Language (Edge & Herrmann, 1977; Healy, 2015; Kegl, 1990; Meir et al., 2007; Oomen, 2017; Sapountzaki, 2005; Winston, 2013). The reported dispreference for stimulus-experiencer verbs in sign languages has at least two possible explanations, namely verb transitivity and lexicalization of thematic roles.

#### Verb transitivity in ASL

Researchers have noted that some predicate meanings do not appear to map onto transitive structures in sign languages, even though similar meanings do so in the spoken language surrounding the Deaf communities.^5^ Examining a small number of verbs from elicited ASL narratives, Edge and Herrmann (1977) noted that certain verbs, e.g., ‘FRIGHTEN’, were not used as transitives (e.g., ‘LISA FRIGHTEN MAYA’, *‘Lisa frightens Maya’*), but rather as one-place predicates (e.g., ‘LISA FRIGHTEN’, *‘Lisa is frightened’*).^6^ Kegl (1990) came to a similar conclusion for psychological verbs in general in ASL, as did later studies of other sign languages (Meir et al., 2007; Oomen, 2017). She specifically notes a lack of stimulus-experiencer psychological verbs in ASL. Findings from experimental studies by Winston (2013), Healy (2015), and Oomen (2017) lend support to the idea that experiencer-objects (which occur in stimulus-experiencer verbs) are dispreferred in sign languages, or at least in ASL and NGT.

Winston (2013) conducted an online study in which participants rated sentences for correctness in ASL, and determined which argument the verb affected. The sentences were intended to have experiencer-subjects (using verbs such as ‘FEAR’ and ‘LOVE’) and experiencer-objects (using verbs such as ‘EMBARRASS’ and ‘INSPIRE’). The findings indicated that constructions involving experiencer-subjects (as in experiencer-stimulus verbs) are preferred over those with experiencer-objects (as in stimulus-experiencer verbs) in ASL. Healy (2015) similarly documents a production preference in ASL for construing events involving psychological verbs with the subject as the experiencer rather than the object. Specifically, Healy shows that when describing events involving verbs like ‘INFURIATE’, ‘IMPRESS’, and ‘FASCINATE’, Deaf native ASL signers^7^ prefer to use the verb intransitively with the experiencer as the subject (e.g., the equivalent of ‘Lisa was frightened [because of Maya]’). This is unlike the transitive stimulus-subject plus experiencer-object construction common in English (e.g., ‘Maya frightened Lisa’).

**Fig. 2:**
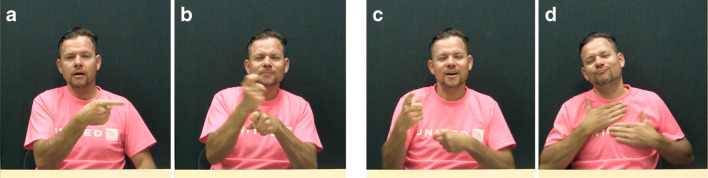
IX-L (**a**) JOKE (**b**) IX-R (**c**) AMUSE (**d**). ‘She (=Leah) (makes) [good funny] jokes. She (=Elsa) (is) amused’

Oomen (2017) found that all psychological verbs in a subset of the NGT sign language corpus (Crasborn et al., 2008, Crasborn & Zwitserlood, 2008) select a subject-experiencer, rather than an object-experiencer. Both Healy (2015) and Oomen (2017) also note that the type of event that is described with psychological verbs is sometimes (for NGT) or generally (for ASL) expressed with bi-clausal structures, with the stimulus and the experiencer occurring in separate clauses (e.g., ‘The girl saw a bear. She was frightened’). Figure 2 shows a signer using such a construction. The signer is explaining that Leah is amusing to Elsa. He signs ‘IX-L GOOD FUNNY JOKE. IX-R AMUSE, which approximately translates to ‘She (Leah) makes good, funny jokes. She (Elsa) is amused’.

#### Lexicalization of thematic roles and the influence of body-anchoring

The aforementioned studies have noted a general tendency to use verbs intransitively in sign languages which are lexicalized as transitive stimulus-experiencer verbs in many spoken languages. However, the findings also include examples where such signed verbs are in fact transitive. Kegl (1990), Healy (2015), and Oomen (2017) all find that some psychological verbs can occur in transitive contexts in ASL and NGT. Nevertheless, the proposed constraint against experiencer-objects appears to hold even when the verb is used transitively. Edge and Herrmann (1977) report that their ASL consultant expressed the meaning ‘Leora angered Vicky’ by signing the experiencer as the subject, and the stimulus as the object, i.e., ‘VICKY ANGER LEORA’. Similarly, in Winston’s (2013) experiment, participants showed substantial disagreement about whether sentences with intended stimulus-experiencer constructions were interpreted as having the syntactic subject (NP1) or the syntactic object (NP2) as the experiencer. Despite this disagreement, these sentences were consistently given relatively high grammaticality ratings, indicating that the signers considered them acceptable ASL structures. Finally, Kegl (1990) notes that the ASL verb ‘SCARE’ appears to work with an object-experiencer, although she suggests that it may not be a psychological verb at all. Rather she proposes that in ‘SCARE’, the subject purposefully acts in a way so as to scare the object. Thus, what these findings suggest is that the type of verb that has stimulus-experiencer structure in languages like English, Spanish, and Dutch resists being put in transitive sentence frames in sign languages. Consequently, when these verbs are used in a transitive construction, the experiencer, not the stimulus, is the subject.

Previous research has proposed that body-anchoring may explain the preference for experiencer-subjects (found in experiencer-stimulus verbs) in sign languages. To reiterate, *body-anchoring* refers to the fact that some signs are obligatorily articulated on or near a particular part of the signer’s body, rather than in neutral signing space. For psychological verbs, body-anchoring has been proposed to foster the understanding that the referent experiencing the emotion or mental state is being mapped onto the signer’s (i.e., producer’s) body. As stated by Edge and Herrmann, ‘[b]ecause these verbs are always articulated on or at the body, they formationally incorporate the experiencer […]’ and thus ‘this argument must appear overtly’ (Edge & Herrmann, 1977: 146). Accordingly, this explanation holds that ASL signers interpret a phrase like ‘LISA FRIGHTEN’ to mean ‘*Lisa is frightened*’ rather than ‘*Lisa frightened (someone)*’, as doing so ensures that the experiencer is the subject. Kegl (1990) similarly notes that experiencers are obligatorily body-anchored. Meir et al. (2007) examine body-anchored verbs (not limited to psychological verbs) that are at least partially iconic. They argue that across these verbs, the signer’s body always correlates with the subject, rather than with a particular thematic role. Under their analysis, the general principles of mapping between thematic and syntactic structure (Fillmore, 1968; Jackendoff, 1990) ensure that the highest-ranking thematic role becomes the subject argument. The highest-ranking role is the agent in agent-patient action verbs, and the experiencer in experiencer-stimulus psychological verbs.

Meir et al. simply note that psychological verbs with stimulus-experiencer structure are not attested in ASL or Israeli Sign Language (ISL). In fact, body-anchoring also provides an explanation for the absence of the stimulus-experiencer structure. Whereas experiencer-stimulus structures allow the body to simultaneously fulfill the roles of subject and experiencer, this is not the case for stimulus-experiencer structures. In the stimulus-experiencer structure, the impetus to interpret the signer’s body as the experiencer conflicts with the interpretation of the body as the subject. Oomen (2017) notes that 70% of the psychological verbs she examined in a subset of the NGT corpus were body-anchored.^8^ She speculates that this might be the case for other sign languages as well.^9^

So far, the evidence appears to suggest that ASL is similar to a language like Mandarin in having psychological verbs lexicalized with experiencer-subjects, but few or no experiencer-objects. In ASL, this state of affairs appears to be linked to a dispreference for using certain psychological verbs transitively, and for lexicalizing verbs with stimulus-experiencer thematic roles. As discussed above, previous studies have shown that stimulus-experiencer verbs reliably elicit NP1-biases in many spoken languages. NP1-biases are also found in agent-patient verbs, but to a much smaller degree and less reliably (Ferstl et al., 2011; Goikoetxea et al., 2008). The absence of verbs with stimulus-experiencer structure in sign languages might lead to a smaller number of NP1-biased IC verbs and in particular to weaker biases towards NP1 compared to NP2. In the two experiments reported below, we assess this possibility in ASL, and also address the question of whether thematic roles predict IC biases in ASL psychological verbs as they do in other languages, despite the modality-specific phenomenon of body-anchoring.

## The present study

Determining the distribution of implicit causality (IC) biases in American Sign Language (ASL) is necessary for future psycholinguistic studies. In addition, understanding implicit causality in a sign language is important given claims that the foundation for this phenomenon is similar across languages and cultures (Hartshorne et al., 2013). This is particularly so in the light of findings from past work in sign language linguistics, namely the suggestion that the visual-manual modality imposes certain restrictions on the mapping between verb semantics and syntax that do not exist in spoken languages (Edge & Herrmann, 1977; Kegl, 1990; Meir et al., 2007). Such restrictions might exert a strong influence on thematic roles and IC biases in ASL.

To discover how IC biases are distributed in ASL, we conducted a norming study with a large set of verbs. Experiment 1 provides a valence norming study, with the goal of defining a set of transitive ASL verbs (videos of the verb signs and transitivity ratings are freely available at the Open Science Framework: https://osf.io/bwjq2/). This set of verbs was used to build the stimuli for Experiment 2, which aimed to discover the direction of the verbs’ IC biases (resulting bias scores can also be found on the Open Science Framework: https://osf.io/bwjq2/). Finally, we analyzed whether thematic role predicts IC biases in a subset of the verbs from Experiment 2 and how this is affected by transitivity preferences and by the modality-specific phenomenon of body-anchoring.

### Experiment 1

#### Participants, materials and procedure

Five Deaf ASL signers rated the acceptability of 292 ASL signs in transitive contexts. With the exception of one participant who learned ASL as young adult, all were native signers.^10^ We constructed the list of 292 signs by using the 305 verbs from the stimuli used by Ferstl et al. (2011). We also added a number of verbs from other sources (Baker-Shenk & Cokely, 1980; Padden, 1988), as well as verbs that we knew to occur in ASL but that do not have (lexical) English translation equivalents. We then evaluated these verbs with the help of a Deaf native ASL consultant. The evaluation procedure was as follows: (1) we discarded verbs that did not have sign equivalents, including certain verbs of sound and hearing (e.g., ‘echoed*’*) that have little relevance in a visual language (Anderson & Reilly, 2002: 86); (2) we kept only one sign where multiple English verbs were glossed with the same ASL sign; and (3) for English verbs whose sign equivalents shared a manual form but differed in mouthing, we kept both signs. In order to avoid excluding potential verbs from norming, we kept all remaining signs. This included glosses that work as more than one part of speech in English (e.g., ‘wow’, ‘value’), even when we assumed that they would only function as one category in ASL. The verb sign videos are available at the Open Science Framework (https://osf.io/bwjq2/).

The rating procedure was as follows. We introduced pictures of two characters, named MAYA and LISA, and we asked the signers to imagine the two names as two referents in a (transitive) frame (i.e., MAYA VERB LISA). Leaving the pictures of the characters visible on the screen, we then showed video clips of the verbs from our list one at a time, creating transitive contexts with different verbs. The signers judged whether the verb was acceptable in the presented transitive frame and noted their answer in a booklet by ticking either a ‘yes’ or a ‘no’ box. The signers were informed that they could have more time for specific verb signs if they needed it. Some signers occasionally made use of this possibility. The task took 45–55 minutes.

#### Results

We calculated an acceptance score for each verb by summing the number of signers who found the verb to be acceptable in the provided context. A score of 5 indicates that all signers found the verb to be acceptable in a transitive context, while a score of 0 indicates that no signer found the verb acceptable in this context. The results (Table 1) show that 5.48% of the verbs were rejected by all signers, and 53.42% were accepted by all signers. Thus, the signers deemed the majority of the verbs fully acceptable.

**Table 1:** Number and proportion of verbs by acceptance score

Score	No. verbs	Proportion of total
0	16	0.055
1	13	0.045
2	24	0.082
3	33	0.113
4	50	0.171
5	156	0.534
Total	292	1.000

Verbs that were accepted by a majority of signers (that is, by at least three out of five signers) comprised 81.85% of the total. Of the verbs, 18.15% (*n* = 53) were deemed unacceptable by three or more signers. Table 2 shows which verbs were rejected by all five signers, by four signers, and by three signers.^11^ The full verb distribution can be found at the Open Science Framework (https://osf.io/bwjq2/).

**Table 2:** Verbs rejected by all five, by four, and by three signers

Acceptance score	% (*N*)	Verb
0	5% (16)	BUG, CHILL, DEPRESS, DISTRESS, ENTERTAIN, FANCY, GOOGLE, GRIEVE, IMPROVE, PREPARE, PROMOTE_2, RELAX, SHAKE, SICKEN, VALUE, WOW
1	4% (13)	AGGRAVATE, BATTLE, CALM, CARESS, CHEAT_2, DEVASTATE, EXHAUST, FLOOR, GALL, MOVE, PUZZLE, SHAME, WEARY
2	8% (24)	ABDUCT, ADD, ANTAGONIZE, ARREST, BAFFLE, BORE, CARRY, COACH, CONCERN, CONSIDER, CURE, DELIGHT, DREAM, EXCITE, FLABBERGAST, HEAL, IMPRESS, INCENSE, PLAY, REFUSE, REPEL, RUMINATE, RUIN, THRILL

### Experiment 2

We used the verbs from Experiment 1 that were deemed acceptable by at least three out of five signers as the basis for the stimuli in Experiment 2. For each of these verbs, we created a sentence fragment to be used in a sentence completion task intended to reveal the verbs’ biases.

#### Participants and procedure

Eight Deaf native signers (mean age: 31.63, range: 22–50) participated in the experiment. After giving informed consent and filling out a background questionnaire, the signers were shown a prerecorded experiment introduction video, which provided instructions in ASL. We gave each signer three practice trials and offered suggestions or clarification where necessary. When the signer felt comfortable with the demands of the task, we turned on the video camera to record their sentences, and they proceeded to the experiment proper, which lasted around one hour.

#### Materials

**Fig. 3:**
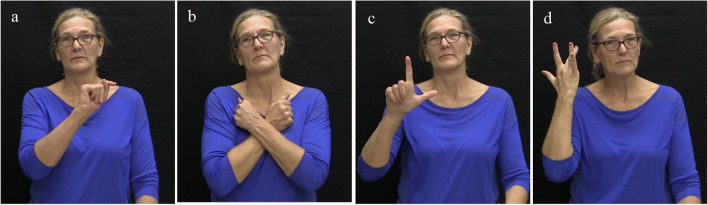
Stimulus example. (**a**) #THOM, (**b**) LOVE, (**c**) #LEAH, (**d**) WHY

We created 239 sentence fragments from the verbs that were found to be acceptable transitives in Experiment 1. The sentence fragments were of the type ‘NP V NP WHY? …’^12^, which is the ASL equivalent of the English ‘NP V NP because … ‘, e.g., ‘#THOM LOVE #LEAH WHY? …’ (Fig. 3).^13^

The English ‘Name Verb Name because …’ sentence frame has been used extensively in research of spoken language verb biases (Garvey & Caramazza, 1974; Koornneef & van Berkum, 2006; Stevenson et al., 1994, among others). In Experiment 2, each NP consisted of a name drawn from a pool of 49 names. Each name consisted of four letters fingerspelled using the hand alphabet. The sentence fragments were presented in randomized order. The participants’ task was to use the sentence fragments as context for free sentence continuations. The instructions were to watch the fragments and complete the sentence with the first continuation that came to mind.

Sentence completion tasks have traditionally been conducted in writing. This has the advantage of giving participants the option of revisiting the context sentence while composing their answer. This ensures that the sentence context remains accessible to participants. Because ASL has no widely accepted written form, the possibility of conducting the experiment in writing was not available to us. We opted instead to ensure participants’ familiarity with the context sentence by asking them to repeat it before providing their own continuation.

#### Coding and evaluation

After collection, a Deaf, native signer coded the participants’ responses for next-mention, that is, which referent they mentioned as the subject in their free continuation. Next-mention was coded as *NP1*, *NP2*, *Both*, *Unclear*, or *Other* (see Examples (3)–(7) below. The next-mention is shown in bold).

(7) **NP1:**

#LOLA-R APPROACH #GALE WHY? **IX-R** NOTICE MANY THINGS CONFLICT APPROACH DISCUSS RESOLVE

‘Lola_i_ approached Gale_j_, because **she**_**i**_ noticed a lot of conflicts, so she_i_ approached (her_j_) to discuss and resolve (them)’

(8) **NP2:**

#KATE ASSIST IX-R #ANDY WHY? **IX-R** BROKE #LEG CRIPPLE

‘Kate assisted Andy, because **he** broke his leg and was unable to walk’

(9) **Both:**

#SAUL BEGUILE #ROSS IX-L WHY? **BOTH** WANT BRING WATCH #CONCERT MUSIC PERFORMANCE

‘Saul beguiled Ross, because **they both** wanted to go watch a concert, a music performance.

(10) **Unclear:**

#TYRA ATTRACT LENA WHY? **Ø** REALLY LIKE IX-L

‘Tyra attracts/is attracted to Lena, because **she** really likes her’

(11) **Other:**

#NOAH SCARE #EARL WHY? #ITS **HALLOWEEN** COSTUME SCARE

‘Noah scares Earl, because it’s **Halloween**, he puts on a costume and scares him

Trials that were accidentally skipped and those where the participant did not provide a meaningful answer (e.g., responded with ‘I don’t know’) were discarded (*n* = 38). We also discarded trials where the participant had understood the verb incorrectly (*n* = 52).^14^ These two categories together amounted to 5% of the total trials.

Independently, another Deaf, native signer coded 50% of the data (four full subjects). The inter-coder agreement was found to be kappa = 0.916. Differences were resolved through discussion where possible; otherwise, the first coder’s choice was retained. The coders were instructed to be conservative and code potential ambiguities as ‘unclear’.

#### Results

We first provide an overview of how participants’ responses were distributed (Table 3). As mentioned above, 5% of trials were discarded from further analysis because they did not provide a meaningful response or contained an incorrect verb meaning. In addition, 40 responses were coded as ‘Unclear’, 135 responses as ‘Both’, and 80 responses as ‘Other’. Responses with these three labels were also excluded from further analysis, amounting to an additional 13% of the total data set. As shown in Table 3, the remaining responses were categorized as NP1-biased (38%) and NP2-biased (44%). A statistical analysis showed a trend towards more NP2 continuations than NP1 continuations (*t*(238) = −1.85, *p* = 0.065).

**Table 3:** Coding of responses

Annotation	N	%	Mean %	SD	Range
No answer	38	.02	.020	4.56	.00–.15
Wrong verb	52	.03	.025	2.10	.01–.04
Unclear	40	.02	.014	3.06	.00–.04
NP1	718	.38	.359	29.32	.21–.55
NP2	849	.44	.483	24.17	.33–.63
Both	135	.07	.072	8.24	.03–.11
Other	80	.04	.028	6.78	.01–.10
Total	1912	1.00	1.001		

We next provide an overview of the biases for individual verbs by calculating a bias score (100*(no.NP1 − no.NP2)/( no.NP1+ no.NP2)) for each verb, collapsing across subject variation.^15^ Across verbs, the calculated bias scores covered the full range of values (*M =* −7.35, SD = 61.27, range = −100 to +100). Similar to the pattern in the continuations overall, the negative mean bias score suggests a preference towards NP2-biased verbs over NP1-biased verbs.

Table 4 bins the bias scores by strength and shows the number of verbs in each bin, excluding verbs for which we had fewer than five responses (*n* = 26). Overall, fewer verbs were biased towards NP1 (41%, *n* = 88) than towards NP2 (54%, *n* = 116). Moreover, fewer verbs were strongly biased towards NP1 (*n* = 34) than were strongly biased towards NP2 (*n* = 55), based on bias scores of above 50 and below −50. Taken together, these results show converging tendencies pointing towards a stronger presence of NP2-biases in the present data set.

**Table 4:** Bias score distribution

Bias	Bias score	No. verbs
NP1	91–100	17
	81–90	0
	71–80	13
	61–70	3
	51–60	1
	41–50	23
	31–40	13
	21–30	8
	11–20	10
	1–10	0
	**Total**	**88**
Equal	0	9
NP2	1–10	0
	11–20	19
	21–30	8
	31–40	8
	41–50	26
	51–60	6
	61–70	6
	71–80	17
	81–90	0
	91–100	26
	**Total**	**116**
	TOTAL	213

### How verb semantics influences implicit causality in ASL

While Experiment 2 suggested that there may be a relatively low proportion of NP1-biased continuations in ASL, it is not clear why this is. It is possible that the observed distribution of IC biases in ASL indicates that thematic roles do not predict IC biases in the manual modality as they do in the spoken modality, possibly because of body-anchoring, and possibly because the relationship between semantic structure and IC bias is still developing in a language as relatively young as ASL. However, given previous research, another likely explanation for the low proportion of NP1-biased verbs is a dispreference in ASL for lexicalizing verbs with stimulus-experiencer structure.

To assess these possibilities, we first asked whether verbs that may have stimulus-experiencer structure in other languages are lexicalized as such in American Sign Language (ASL). We focused on the verbs in our stimuli for which the glosses are classified as stimulus-experiencer verbs in Ferstl et al. (2011).^16^ We first examined whether these verbs are more likely to be intransitive, rather than transitive, as compared to verbs from other categories. We did this by analyzing the verbs that were rejected by the majority of signers in Experiment 1. This analysis shows that 55% (*n* = 53) of the rejected verbs had glosses that were classified as stimulus-experiencer by Ferstl and colleagues. Nevertheless, 58% of all verbs with stimulus-experiencer glosses (*n* = 69) were judged to be acceptable by three or more signers. As these verbs were included in Experiment 2, we can examine the distribution of their bias scores. This examination helps us assess whether these verbs were consistently biased in one direction over the other. The analysis revealed bias scores distributed on a continuum from strongly NP2-biased (scores of −100 to −66: CHEER, COMFORT, ANNOY, AMAZE, DISGUST, INTEREST, AMUSE, FASCINATE, ENTICE), to not clearly biased (scores of −25 to 25: e.g., ENCOURAGE, TEMPT, SCARE, CONFUSE), to strongly NP1-biased (scores of 100 to 66: ATTRACT, HARASS, INSULT, CHARM, FLATTER, EMBARRASS, PAIN).

**Fig. 4:**
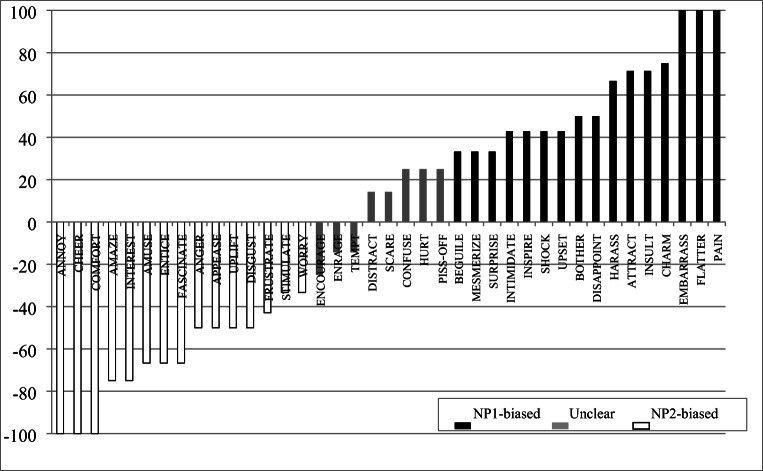
Distribution of bias scores for verbs lexicalized as stimulus-experiencer in English

There is an even distribution of NP2-biased (*n* = 15) and NP1-biased (*n* = 16) verbs (Fig. 4). The fact that the majority of these verbs show clear biases suggests overall agreement among signers about the bias of individual verbs rather than a pattern based on ideolectal differences. On the other hand, the distribution encompasses both types of biases, suggesting that this group of verbs is not homogenous in ASL.

We next used the sentence contexts produced by the signers to determine the thematic roles most frequently occurring with each of the potential stimulus-experiencer verbs (Table 5). We used the transcription of each response (excluding those labeled as N/A or wrong verb) to categorize the thematic roles occurring with the verb as AgP, StimExp, ExpStim, Unclear, or AgP/StimExp (ambiguous between Agent-Patient and Stimulus-Experiencer). Aggregating over signers, we then assessed which thematic role constellation was most frequent for each verb. If there was no one constellation used by a majority of signers, we coded the predominant thematic role as Unclear. This analysis revealed that the split in the bias direction within this category of verbs is largely accompanied by a split in thematic structure. Specifically, as a group the NP1-biased verbs in the current data set are more frequently lexicalized with stimulus-experiencer structure compared to other structures. Eight out of 16 verbs occurred more often with stimulus-experiencer structure than with any other structure, including ‘INSULT’, which was ambiguous between stimulus-experiencer and agent-patient structure.

**Table 5:** Biases and thematic structures in the stimulus-experiencer category

Verb	Bias	Bias strength	Predominant thematic role	Frequency of thematic role
CHEER	NP2	−100 to −75	AgP	7/7
ANNOY			ExpStim	8/8
AMAZE			ExpStim	7/8
DISGUST			ExpStim	6/8
INTEREST			ExpStim	8/8
COMFORT			StimExp/AgP	8/8
AMUSE		−74 to −33	ExpStim	5/7
FASCINATE			ExpStim	7/8
ANGER			ExpStim	7/8
FRUSTRATE			Unclear	3 ExpStim; 3 StimExp
WORRY			ExpStim	6/8
ENTICE			AgP	6/7
APPEASE			StimExp/AgP	7/7
UPLIFT			AgP	5/8
STIMULATE			StimExp/AgP	3/5
ENRAGE	Unclear	−32 to 32	ExpStim	5/7
TEMPT			StimExp	4/7
DISTRACT			AgP	4/8
SCARE			StimExp	5/8
CONFUSE			StimExp	5/8
PISS-OFF			ExpStim	5/8
ENCOURAGE			StimExp/AgP	8/8
HURT			StimExp/AgP	4 StimExp; 4 AgP
MESMERIZE	NP1	33–74	Unclear	5/8
SURPRISE			StimExp	7/7
INSPIRE			StimExp	5/8
SHOCK			StimExp	6/8
UPSET			ExpStim	4/7
DISAPPOINT			StimExp	6/8
BEGUILE			Unclear	4 AgP; 4 Unclear
BOTHER			AgP	8/8
INTIMIDATE			AgP	8/8
ATTRACT		75-100	StimExp	5/8
EMBARRASS			StimExp	7/8
HARRAS			AgP	7/8
CHARM			AgP	7/8
INSULT			StimExp/AgP	6/8
FLATTER			AgP	6/7
PAIN			StimExp	6/8

**Fig. 5:**
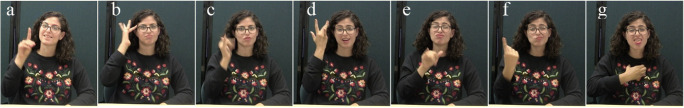
Depiction of Example (12). (**a**) #DANA, (**b**) ANNOY, (**c**) #JADE, (**d**) WHY, (**e**) #JADE, (**f**) ALWAYS, (**g**) COMPLAIN

The reverse was true for the NP2-biased verbs, which were most frequently lexicalized with experiencer-stimulus structure in the current data. In this group, eight out of 15 verbs occurred with experiencer-stimulus structure more frequently than they occurred with any other structure. Despite their glosses suggesting that they should be stimulus-experiencer verbs, some verbs, such as ‘ANNOY’ and ‘AMAZE’, in fact had experiencer-stimulus structure. This suggests that when they are used in transitive contexts (‘MAYA ANNOY LISA’), they are understood along the lines of ‘Maya is annoyed with/by Lisa’ rather than ‘Maya annoys Lisa’ (Example (12), Fig. 5). In sum, in this group of potential stimulus-experiencer verbs, those that are biased towards NP2 tend to also have experiencer-stimulus structure. This is in line with previous research findings, namely that experiencer-stimulus verbs tend to be NP2-biased. Consequently, although the direction of bias is unexpected compared with the English verb matching the gloss, the direction of the bias in these ASL verbs is nevertheless predictable based on thematic structure.

Within the NP2-biased verbs as well as the NP1-biased verbs, we also find examples with neither stimulus-experiencer nor experiencer-stimulus structure. In the NP2-biased group, some signers used the first NP in a more agentive role instead, such that the verbs are best interpreted as agent-patient verbs (Example (13)).

(12) #DANA ANNOY #JADE WHY? #JADE ALWAYS COMPLAIN ANNOY
  ‘Dana is annoyed with Jade, because Jade always complains. [Dana is] annoyed’

(13) #TROY CHEER #NORA WHY? IX-R SCORE

‘Troy cheered Nora, because she scored’

Although stimulus-experiencer occurred as the predominant role more often than did any other thematic structure, we also found a number of other structures for NP1-biased verbs. Importantly, many involved the agent-patient thematic roles. Some structures were ambiguous between agent-patient and stimulus-experiencer structures. In other instances, a verb that is lexicalized as a stimulus-experiencer verb in other languages was clearly used as an agent-patient verb in this sample of ASL. This is similar to what Kegl (1990) reported for ‘SCARE’. In the examples below, the subject is an agent intentionally causing something to happen to the patient in (14), and the subject is a potentially unwitting stimulus for an emotion in the experiencer in (15).

(14) #MARA HURT #GALE WHY #MARA MAD HIT HIT #GALE

‘Mara hurt Gale, because Mara got angry and hit Gale’

(15) #MARA HURT #GALE WHY IX-L DECIDE CUT DONT-WANT
  IX-R IN POSS-L LIFE ANY MORE
  ‘Mara hurt Gale, because she decided to cut her off, she didn’t want her in her life anymore’

All told, there are 16 actual stimulus-experiencer verbs within the group of verbs with stimulus-experiencer glosses (including six with ambiguous stimulus-experiencer/agent-patient structure). This corresponds to 7.5% of the 213 verbs in Experiment 2 for which we calculated bias scores. This suggests a low proportion of stimulus-experiencer verbs in ASL overall and begs the question whether this is a result of body-anchoring. In the final analysis, we therefore investigated whether certain thematic roles were more or less likely to occur in body-anchored verbs in the current data set.

Table 6 shows how body-anchoring interacts with bias and actual thematic role in the potential stimulus-experiencer verbs in the current study. These data do not suggest that ASL verb bias is a function of body-anchoring. All experiencer-stimulus verbs are body-anchored, and the majority of them are NP2-biased (8/11). Most stimulus-experiencer verbs are body-anchored as well, and most of the body-anchored verbs are also NP1-biased (5/7). Thus, there is no evidence in the present data set that the experiencer is the preferred referent in continuations of sentences containing body-anchored verbs.

**Table 6:** Body-anchoring and thematic roles within the assumed stimulus-experiencer category

Thematic structure	No.	BIAS	VERBS	
			Body-anchored	Not body-anchored
ExpStim (*n* = 11)	1	NP1	UPSET	
	8	NP2	AMAZE, AMUSE, ANGER, ANNOY, DISGUST, FASCINATE, INTEREST, WORRY	
	2	Unclear	ENRAGE, PISS-OFF	
StimExp (*n* = 10)	7	NP1	DISAPPOINT, EMBARRASS, INSPIRE, SHOCK, SURPRISE	ATTRACT, PAIN
	0	NP2		
	3	Unclear	CONFUSE, SCARE, TEMPT	
StimExp/AgP (*n* = 6)	1	NP1		INSULT
	3	NP2		APPEASE, COMFORT, STIMULATE
	2	Unclear		ENCOURAGE, HURT
AgP (*n* = 9)	5	NP1		BOTHER, CHARM, FLATTER, HARASS, INTIMIDATE
	3	NP2	CHEER	ENTICE, UPLIFT
	1	Unclear	DISTRACT	
Unclear (*n* = 3)	2	NP1	MESMERIZE	BEGUILE
	1	NP2	FRUSTRATE	
	0	Unclear		

The distribution of verbs in Table 6 also does not suggest a strong role for body-anchoring in determining whether verbs are lexicalized with stimulus-experiencer or experiencer-stimulus structure. If body-anchoring required the experiencer to appear as the first argument, we would not expect to find body-anchored verbs with stimulus-experiencer structure.

**Fig. 6:**
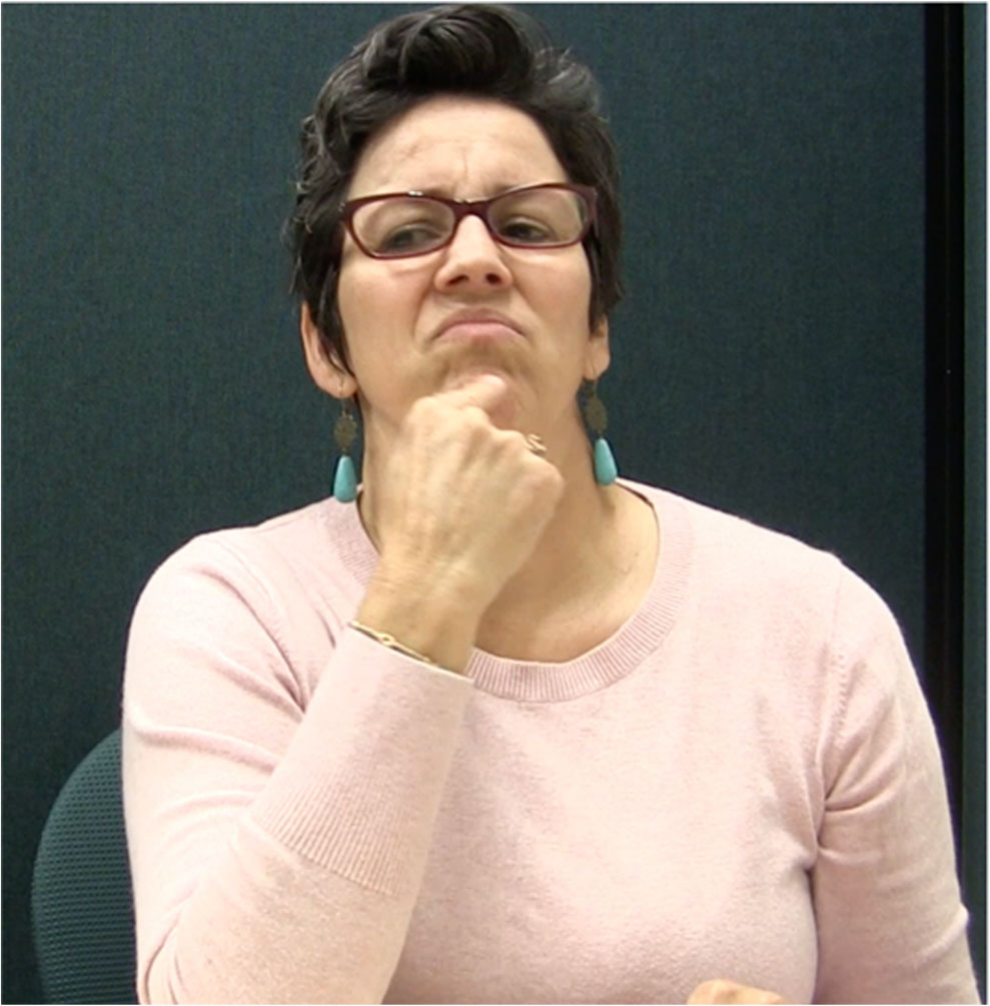
DISAPPOINT

**Fig. 7:**
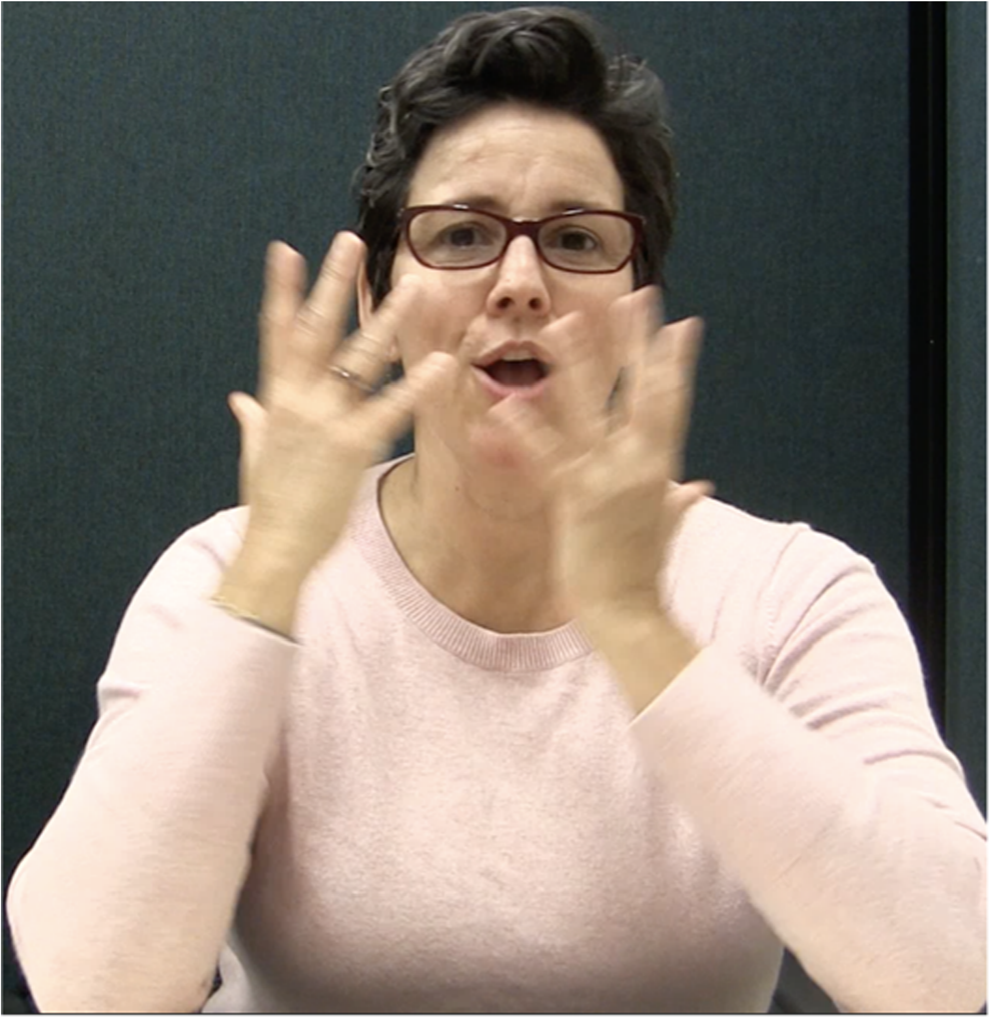
EMBARRASS

**Fig. 8:**
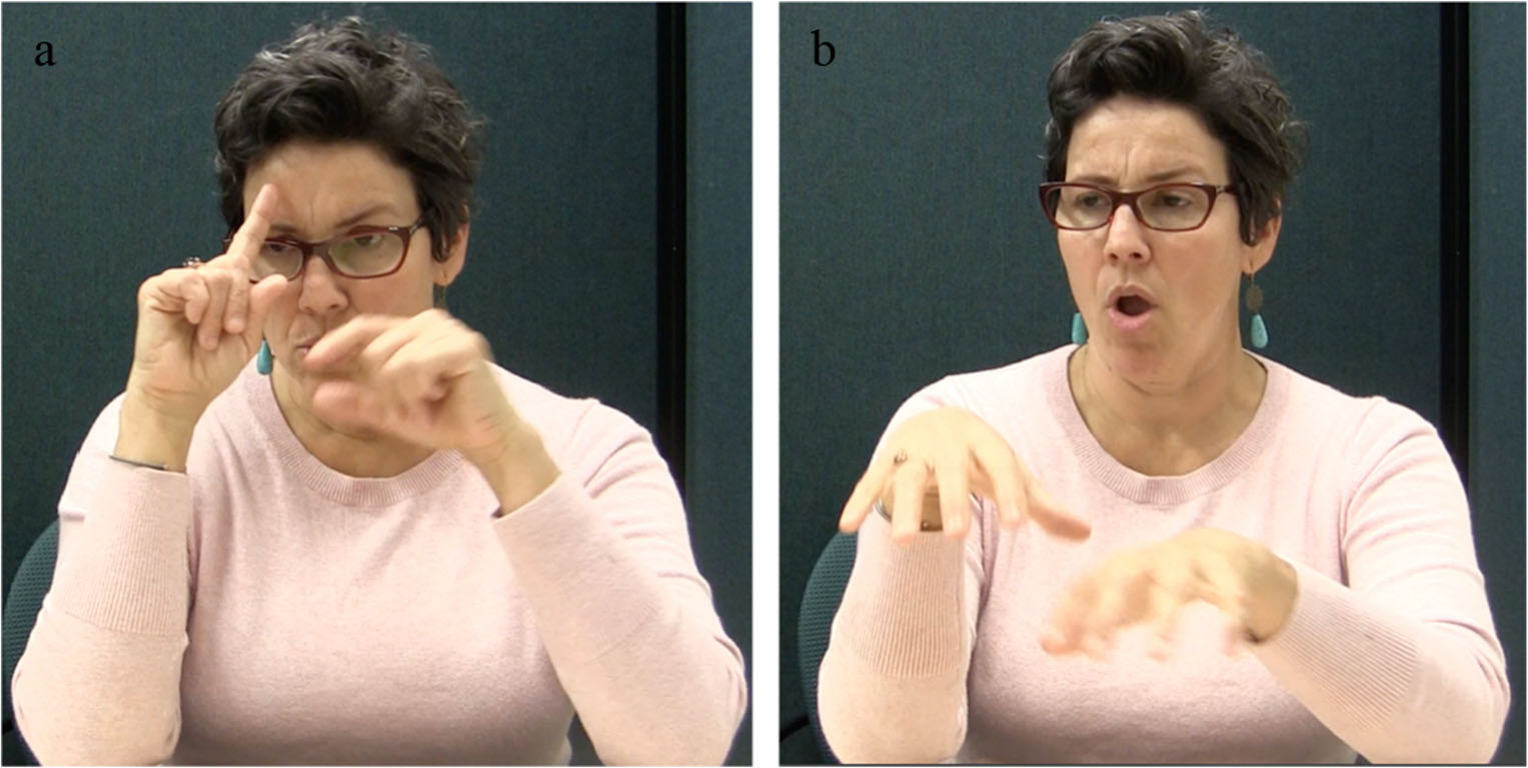
SHOCK, beginning (panel a) and end (panel b) of sign.

However, we do find such verbs (e.g., ‘DISAPPOINT’, ‘EMBARRASS’, and ‘SHOCK’, Figs. 6, 7 and 8). As Table 6 shows, eight verbs were body-anchored out of the 16 verbs that had stimulus-experiencer structure or were ambiguous between stimulus-experiencer and agent-patient structure. While all of the verbs that have experiencer-stimulus structure are in fact body-anchored (11/11), nearly all of the actual stimulus-experiencer verbs are body-anchored as well (7/10). Therefore, the results of the present study do not support the idea that the phonological feature of body-anchoring in some signs is the primary predictor in ASL for whether a verb will be lexicalized as a stimulus-experiencer verb or not.

On the other hand, the verbs which are ambiguous between an agent-patient and a stimulus-experiencer reading are *not* body-anchored. The pattern of stimulus-experiencer and experiencer-stimulus verbs being mostly body-anchored suggests a correlation between potential psychological verbs being articulated on the body and being interpreted as involving experiences or emotions rather than actions.^17^

## Discussion

In two experiments, we examined the distribution of implicit causality (IC) biases in 239 verbs from American Sign Language (ASL). We also examined the relationship between thematic role and IC bias in a group of ASL verbs, namely those lexicalized as stimulus-experiencer verbs in English. IC biases are the foundation for much psycholinguistic research, and the present study is the first to document these in ASL and provide norming data that can be applied in future studies. Because IC verbs have not previously been studied in sign languages, we compared our ASL results with English as a way to emphasize where and how the signed modality might differ from the spoken one.

### IC biases in ASL and English

The IC biases in our sample are distributed across the full range of possible values, from strongly NP1-biased to strongly NP2-biased. This is similar to what has been found for other languages. However, the results also suggest differences between ASL and English. Specifically, we found a trend towards more NP2 continuations as opposed to the trend towards more NP1 continuations in English reported by Ferstl et al. (2011). Moreover, in Ferstl et al. (2011) there were more NP1-biased (*n* = 88) than NP2-biased (*n* = 73) among the strongly biased verbs. The opposite was true in the present study, where the number of verbs strongly biased towards NP2 was higher than that of verbs strongly biased towards NP1. However, this difference should be considered in the light of variation between data sets. Two more recent studies of biases for larger samples of verbs reported an overall bias towards the object in English, although to different degrees (Hartshorne et al., 2015; Hartshorne & Snedeker, 2012). This indicates that the overall preference for bias direction varies as a function of the verbs included in the test. In the present study, we based our stimuli on the verbs used in Ferstl et al. (2011). However, the final data set in Experiment 2 used just over half of the verbs (*n* = 125) used by Ferstl and colleagues. An additional 47 verbs from Ferstl et al. were included in Experiment 1 but were shown to be unacceptable in transitive contexts. The majority of the excluded verbs had glosses that are expected to be NP1-biased in English.

The use of glosses to represent ASL signs also introduces variation. We based our ASL verb data set on verbs used in English by Ferstl et al. (2011). Thus, for each English verb, we looked for an ASL sign with the same meaning. Thus, some stimulus signs could have been glossed differently using a different approach. ASL glosses are English words, and therefore it is easy to assume that an ASL sign is equivalent in meaning to the English word used for the gloss. In fact, there is limited consensus among both signers and sign language researchers in how to choose which English word(s) to use as the gloss for a sign. For now, the choice is, to some extent, arbitrary. This is essentially the problem of determining what the best translation is between words in any two languages. As noted by Hartshorne et al. (2013), this problem poses a challenge for researchers trying to determine whether the same verbs exhibit the same re-mention biases across languages.

Nevertheless, the trend in our results is towards fewer and less strongly biased NP1-biased verbs in the current data set, compared to NP2-biased verbs, despite the fact that our data set was largely based on the verbs used in Ferstl et al., who found more NP1 than NP2 continuations. The trend towards preferring NP2 continuations, we attribute in part to the observed preferences for lexicalizing thematic roles in ASL verbs. This is in line with previous work on spoken languages that has found that verb semantics, including thematic role, predicts the direction of a verb’s IC bias, with stimulus-experiencer verbs exhibiting particularly robust NP1-biases.

### The existence of stimulus-experiencer verbs in ASL

The signers’ use of thematic roles in Experiment 2 show that the stimulus-experiencer structure does exist in ASL. This is a somewhat surprising finding, given previous indications in the sign language literature that this structure is not attested. Yet, stimulus-experiencer verbs seem to be rarer in ASL than in languages like English. Experiment 1 offered partial support for the suggestion that the rarity of stimulus-experiencer verbs is partially due to the fact that verbs lexicalized as such in other languages tend not to occur as syntactic transitives in ASL. Our results show that the majority of verbs that were unacceptable in transitive contexts were verbs lexicalized as stimulus-experiencer verbs in English according to Ferstl et al. (2011). However, it is important to note that the results of the transitivity rating in the current study were not categorical. In Experiment 1, there was agreement about the transitivity status of 59% percent of the signs. Conversely, there was at least some disagreement about the remaining 41% of signs. There are a number of possible reasons for the high degree of disagreement including the variation in the signing community due to influence from English, regional and dialectal differences, and different ages of first language acquisition and the large proportion of second language signers. In addition, the task we used to obtain transitivity ratings may produce different ratings compared to a more traditional grammatical judgment task in which participants are presented with complete sentences and judge whether they are grammatical. We decided not to use the more traditional task in an effort to avoid coercing intransitive verbs into transitive sentences, which signers might deem acceptable because of exposure to and experience with English-influenced signing. However, with the paradigm used in the present study, signers may have judged whether they themselves would use a particular sentence, rather than its acceptability when signed by someone else. This could lead to more gradient ratings compared to the more traditional task.

The results of Experiment 2 showed that many of the potential stimulus-experiencer verbs that were acceptable as transitives in Experiment 1 had experiencer-stimulus structure in ASL.^18^ In our sample, the proportion of verbs with stimulus-experiencer structure was 7.5% (16/213). By comparison, the sample of English verbs studied by Ferstl et al. (2011) contained 36% (110/305) that were lexicalized as stimulus-experiencer verbs. This suggests a rather low rate of stimulus-experiencer verbs in ASL overall, consistent with previous claims. Ferstl et al. (2011) found that their stimulus-experiencer verbs were almost exclusively NP1-biased. Therefore, the low number of these verbs in ASL is likely to have affected the distribution of IC biases in the language, specifically the amount of NP1- compared to NP2-biased verbs. Moreover, the strength of NP1-bias in the actual stimulus-experiencer verbs in ASL does not appear to be as great as in other languages. Of the 16 verbs, which were used either primarily with stimulus-experiencer structure or were ambiguous between stimulus-experiencer and agent-patient structure, only seven were clearly NP1-biased in our results. Moreover, we found no evidence that body-anchoring in ASL changes the relationship between thematic roles and direction of the IC bias.

### Explaining variation in lexicalization of thematic role in ASL

Previous research has offered body-anchoring as an explanation for the lack of stimulus-experiencer verbs in ASL. It has been argued that the reason why psychological verbs are incompatible with stimulus-experiencer structure is because these verbs often have the phonological feature of being body-anchored and thus require the body of the signer to simultaneously function as the subject argument and the experiencer. While our results revealed that a number of body-anchored verbs with potential stimulus-experiencer structure were in fact experiencer-stimulus verbs in the present study, they also showed the existence of body-anchored verbs with stimulus-experiencer structure. Consequently, body-anchoring alone cannot account for the scarcity of the stimulus-experiencer thematic structure in ASL. Thus, the small proportion of stimulus-experiencer verbs in ASL is not necessarily indicative of a difference between the visual-manual and the aural-oral modality. In fact, Mandarin is also reported to disprefer the stimulus-experiencer structure. This could suggest that the lexicalization pattern observed in ASL is unrelated to any properties of its modality. Another possibility, however, is that the same system can occur in spoken and signed languages as a result of different factors or trajectories. In their work on body-as-subject, Meir et al. (2007) make a pertinent prediction. They suggest that all sign languages should initially have verbs where the body is understood as the subject, and that over time, other verb classes may develop, such as agreeing verbs that move in space without contact to the body (Meir et al., 2007: 534). If the overall association between body and subject weakens as such verbs develop, then such a diachronic change might eventually allow for a dissociation between subject and body in body-anchored verbs as well. This could result in a system like the one we observe in the present study, where stimulus-experiencer verbs are attested but infrequent. If so, it is possible that frequency plays a role in determining whether a verb will be interpreted as a stimulus-experiencer or an experiencer-stimulus verb. Although much progress has been made towards producing a frequency database in ASL (see Caselli et al., 2017; Mayberry et al., 2014), existing resources were insufficient for our stimulus items to be analyzed as a function of frequency. Thus, while the ASL and Mandarin systems may look nearly identical on the surface, they may be products of drastically different processes.

An ongoing shift in the association between body and subject could also help explain why we observed variation among signers in the thematic roles used with individual verbs. Much of the disagreement in thematic role use was related to variation in using verbs with stimulus-experiencer or agent-patient structure, but there were also cases where signers disagreed on whether to interpret the same verb as having stimulus-experiencer or experiencer-stimulus structure. Such disagreements could potentially cause communicative problems. If ‘LISA ANNOY MARY’ means that Lisa annoys Mary for one person but means that Lisa is annoyed with Mary for another, then there is potential for misunderstanding. Whether or not such cases represent true ambiguity within individual verbs is an important question. It is possible that many signers are willing to accept stimulus-experiencer structures from other signers but tend to avoid them in their own signing. Healy (2015) looked at ASL production and found no instances of the stimulus-experiencer structure. By comparison, Winston (2013) had signers rate prerecorded ASL utterances. She found that although sentences with experiencer-subjects were rated as more acceptable than those with experiencer-objects, the latter type was seen as acceptable in ASL. The findings of the present study are in line with Winston’s findings. We asked signers to judge the acceptability of structures and then to copy and complete a number of sentence fragments, some of which contained potential stimulus-experiencer verbs. Thus, some of the disagreements may be related to differences in willingness to accept constructions that do not occur in one’s own signing. Another possibility is that there is a set of verbs that can be used with different mappings between thematic roles and syntax, either by individual signers (i.e., the same signer can use the same verb with either experiencer-stimulus or stimulus-experiencer structure) or by the community (i.e., some signers use a given verb with experiencer-stimulus structure while others use it with stimulus-experiencer structure). In both cases, the ambiguity resulting from such variation is likely resolvable in context. Deaf signers are extremely adept at communicating in the face of variation. The signing community is made up of Deaf and hearing individuals who acquired their sign language at a range of ages and are bi- or multilingual to different degrees; only a small proportion of Deaf individuals learned a sign language from birth. All of these factors introduce variation. It is possible that socio-linguistic factors including English influences on one’s signing can indicate the types of thematic structures that are likely to be used. Most Deaf signers know some amount of English, and many are highly proficient sign-text bilinguals. Future work should explore whether there is a correlation between English skills and frequency of use on the one hand, and use/acceptance of the stimulus-experiencer structure in ASL on the other. It is important to note, however, that despite this wide variation in language experience among ASL signers, the participants in the present study showed substantial agreement in their ratings.

## Conclusions and future directions

The current study presented norming data for implicit causality (IC) biases in American Sign Language (ASL) verbs and examined whether differences related to lexicalization of thematic role and phonological restrictions in ASL are correlated with a different distribution of IC biases compared to many spoken languages.

To the best of our knowledge, the present study is the first to offer norming data for implicit causality biases in ASL verbs. Almost 300 verbs were assessed for transitivity, resulting in 239 transitive verbs being tested for their IC bias in a sentence continuation experiment. We found that IC biases in ASL cover the full range of biases, from strongly NP1-biased to strongly NP2-biased, and that ASL may have a smaller proportion of NP1-biased verbs compared with some, but not all, spoken languages. Because past studies have shown that verb semantics predicts direction of IC bias, we hypothesized that the distribution of verb biases we observed in ASL could be linked to the claim from previous literature that ASL lacks stimulus-experiencer verbs. Our results revealed a very small number of verbs with stimulus-experiencer structure in ASL. A number of those verbs lexicalized as stimulus-experiencer verbs in English were interpreted in ASL as having experiencer-stimulus structure instead, and consequently many of these verbs were biased towards NP2 rather than NP1. The fact that many NP1-biased verbs in spoken languages owe their bias to their stimulus-experiencer structure led us to conclude that the limited number of true stimulus-experiencer verbs impacts the distribution and overall number of NP1-biased verbs in ASL. This is an important finding for future experimental psycholinguistics studies of ASL. Overall, our analyses provide some evidence that thematic role predicts IC bias in ASL. Finally, we did not find that the phonological feature of body-anchoring predicts whether a verb will be lexicalized as a stimulus-experiencer verb, contrary to the claims in previous research.

The conclusions of the present study are limited by the relatively low number of participants compared to studies of IC biases in spoken languages. Although we believe our IC bias norming results to be representative for ASL in general, our thematic role analyses examined only a subset of verbs and are therefore more susceptible to characteristics of our signer sample. Future work should confirm these results by obtaining IC biases from a larger and more geographically varied group of signers and investigate whether variation among languages in the distribution of IC biases is indicative of systemic differences that may affect processes such as pronoun resolution. In addition, future work should expand on the present results by studying ASL thematic roles in a larger sample of verbs and pursuing reasons for variation of thematic role lexicalization in ASL, such as verb frequency and variation in English proficiency and English use.

## Footnotes

1 Note that this does not mean it has to be the signer who is experiencing the emotion. Sign languages like ASL allow for the signer’s body to represent another referent in discourse (see Metzger, 1995).

2 We thank Marloes Oomen for this suggestion

3 Although note that bias directions can change with type of sentence connective (Ehrlich, 1980; Stevenson et al., 1994; Pickering & Majid, 2007; Fukumura & van Gompel, 2010; Koornneef & Sanders, 2013) and discourse context (Kehler et al., 2008, Rohde & Kehler, 2014).

4 We use the terms ‘stimulus-experiencer verbs’ and ‘object-experiencer verbs’ interchangeably in the remainder of this paper. The same goes for ‘experiencer-stimulus verbs’ and ‘experiencer-subject verbs’.

5 Deaf with an uppercase “D” is used to denote individuals who identify as culturally deaf, that is, who are members of the Deaf community and who, usually, use a natural sign language.

6 Such predicates are sometimes glossed as adjectives (Klima & Bellugi, 1979), although there is debate about which signs should be considered adjectives and indeed whether ASL in fact has adjectives as a part-of-speech (Klima & Bellugi, 1979; Loos, forthcoming).

7 We use the term ‘native signer’ to mean an individual who acquired a sign language from birth.

8 The proportion increases to nearly 95% if signs where the signer’s hands represents hands or legs/feet of a referent are included as well as those where the head or body is the place of articulation.

9 Note also that there is a relationship between body-anchoring and whether or not agreement is marked on the verb. Not all signed verbs mark agreement (Friedman, 1975; Padden, 1988). Those that do use modifications in hand orientation or the movement of the hand(s) though space to mark verb arguments. So-called plain verbs do not mark agreement. Most body-anchored verbs are plain verbs. Because they are articulated on the body, they are not free to move in space in order to mark subject and object arguments.

10 Due to an experimenter error, age information was not collected

11 The variation in transitivity ratings is discussed in section 4.2

12 ASL makes use of a number of question words, including ‘WHY’ to create so-called rhetorical questions. These are truth-conditionally equivalent to declarative sentences (Caponigro & Davidson, 2011), and the question words have coordinating, rather than subordinating function. The ASL connective ‘WHY’ is therefore functionally equivalent to English ‘because’ in the contexts used in the present study.

13 The hash-key notation indicates that a word is spelled with the manual alphabet (as opposed to being a lexical sign).

14 The proportion of misunderstood verbs can likely be attributed to the fact that a number of ASL verbs have multiple meanings that are usually disambiguated by context, which was not present in our information-lean stimulus structure.

15 no.NP1 and no.NP2 is the total number of NP1 and NP2 continuations for each verb.

16 This approach does not guarantee that we have included all verbs lexicalized as stimulus-experiencer verbs in our ASL data set. However, previous studies have found that thematic role structures such as experiencer-stimulus behave in sign languages as expected from English. This suggests that only the stimulus-experiencer structure is systematically different. We therefore believe it unlikely that we will find many stimulus-experiencer verbs among the verbs whose translation equivalents belong in other thematic role classes. Note also that because there is no consensus on sign glossing, some signs could alternatively be glossed with English words that are not necessarily stimulus-experiencer verbs.

17 While the results do not suggest that body-anchoring determines whether a verb is lexicalized as experiencer-stimulus vs. stimulus-experiencer, the distribution in Table 6 does suggest a possible relationship between thematic role and visual articulatory properties of the verb. Specifically, in the present study, verbs that are body-anchored do not have readings that are ambiguous between agent-patient and stimulus-experiencer thematic roles. It appears that only verbs that are not body-anchored have this ambiguous reading as an option. This suggests that there may be a link between articulatory properties and interpretation of verbs, such that when verbs are articulated on the body, they are interpreted as involving experiences or emotions rather than actions, and vice versa.

18 More than half the verbs lexicalized as stimulus-experiencer verbs in English were acceptable as transitives. This is in line with findings from Winston (2013), who found that translation equivalents of eight English stimulus-experiencer verbs were acceptable as transitives in ASL, although signers varied in the thematic roles they applied to the verb. Winston tested the verbs ‘EMBARRASS’, ‘SCARE’, ‘SURPRISE’, ‘INSPIRE’ (which have stimulus-experiencer structure in the present study), ‘WORRY’, ‘AMUSE’, ‘FASCINATE’ (which have experiencer-stimulus structure in the present study), and ‘BORE’ (in the present study, ‘BORE’ was deemed unacceptable in a transitive context in Experiment 1 and thus not included in Experiment 2). Although she does not provide results for the individual verbs, Winston’s overall finding is similar to what we find in the present study.
